# Serum Vitamin D Level and Disease Activity in Patients With Systemic Lupus Erythematosus

**DOI:** 10.7759/cureus.93853

**Published:** 2025-10-05

**Authors:** Aye Thin Zar Oo, Wint Thandar Oo, Kyaw L Tun, Aung K Myint, May Thu Zar Aye, Nandar E Khin, Khin Pyae Sone, Pyae Sonn

**Affiliations:** 1 Internal Medicine, University of Medicine, Mandalay, Mandalay, MMR; 2 Medicine, University of Medicine 1, Yangon, Yangon, MMR; 3 Medicine, Barking, Havering and Redbridge NHS Foundation Trust, London, GBR; 4 General Medicine, University of Medicine, Mandalay, Mandalay, MMR; 5 Internal Medicine, Mandalay General Hospital, Mandalay, MMR

**Keywords:** autoimmunity, biomarker, disease activity, myanmar, systemic lupus erythematosus, vitamin d

## Abstract

Introduction: Systemic lupus erythematosus (SLE) is a chronic autoimmune disease with variable clinical manifestations and disease activity. Emerging evidence suggests that vitamin D may play a role in immune regulation and disease pathogenesis. This study aimed to investigate the association between serum vitamin D levels and disease activity in patients with SLE at Mandalay General Hospital.

Methods: A cross-sectional study was conducted involving 60 patients diagnosed with SLE. Disease activity was assessed using the Systemic Lupus Activity Measure (SLAM) score. Serum vitamin D levels were measured using the enzyme-linked immunosorbent assay (ELISA) at the National Health Laboratory, Mandalay. Patients were categorized into active and inactive disease groups based on SLAM scores, and intergroup comparisons were made.

Results: Among the 60 patients, the majority (43.3%) were aged 21-30 years, with a mean ± SD age of 25.77 ± 7.98 years. Female patients predominated (98.3%; female-to-male ratio 59:1). The mean SLAM score was 9.25 ± 2.94. Common clinical features included anaemia (56.7%), skin rash (33.3%), mucosal ulcers (16.7%), arthritis (16.7%), serositis (11.7%), vasculitis (11.7%), and neuropsychiatric lupus (5%).

Patients with active disease had significantly lower serum vitamin D levels (12.2 ± 1.36 ng/mL) compared to those with inactive disease (26.1 ± 10.22 ng/mL; t = 5.55, p < 0.0001). A moderate inverse correlation was observed between SLAM scores and vitamin D levels (Pearson’s r = -0.51, p < 0.0001), suggesting a clinically meaningful association between lower vitamin D levels and higher disease activity.

Conclusion: This study demonstrates a moderate inverse relationship between serum vitamin D levels and SLE disease activity. These findings suggest that vitamin D may serve as a potential biomarker for disease monitoring. However, due to the cross-sectional design and relatively small sample size, causality cannot be established. Further longitudinal studies are recommended.

## Introduction

Systemic lupus erythematosus (SLE) is a complex autoimmune disease characterized by immune-mediated damage to multiple organs and tissues [1]. Its pathogenesis involves the production of autoantibodies and immune complexes, leading to chronic inflammation and tissue injury. Globally, the prevalence of SLE varies, influenced by ethnic, geographic, and environmental factors. In the United States, the prevalence is estimated at 124 per 100,000 population, with a strong female predominance and peak onset between 15 and 40 years of age [2]. In Myanmar, hospital-based records from Mandalay General Hospital documented 233, 216, and 253 admissions for SLE in 2016, 2017, and 2018, respectively.

Due to its heterogeneous clinical presentation and unpredictable course, diagnosing and managing SLE remains a significant challenge. Monitoring disease activity is essential for guiding treatment, assessing response, and minimizing irreversible organ damage. Several validated scoring systems are used for this purpose, including the SLE Disease Activity Index (SLEDAI), the British Isles Lupus Assessment Group index (BILAG), and the Systemic Lupus Activity Measure (SLAM). Among these, SLAM was selected in this study for its practicality in clinical settings, especially in resource-limited environments, and its inclusion of patient-reported symptoms alongside clinical and laboratory parameters, which offers a more comprehensive reflection of disease activity in real-world practice [3,4].

Vitamin D has gained attention for its immunomodulatory properties and potential role in the pathogenesis of autoimmune diseases, including SLE [5,6]. It influences both innate and adaptive immunity by modulating T-cell responses, inhibiting dendritic cell maturation, and suppressing B-cell proliferation [7,8]. Several studies have demonstrated an inverse relationship between serum vitamin D levels and SLE disease activity, suggesting that vitamin D deficiency may contribute to increased disease flares and severity [9]. However, the literature remains inconsistent, with some studies reporting no significant association, possibly due to differences in study design, population characteristics, disease activity indices used, and seasonal or geographic variation in vitamin D levels [10].

Vitamin D deficiency is common even in sun-rich regions due to factors such as limited sun exposure, skin-covering clothing, urban indoor lifestyles, and low dietary intake [11]. In Myanmar, no previous studies have examined the relationship between vitamin D status and SLE disease activity. Given the influence of geographic, ethnic, and cultural factors on both vitamin D metabolism and autoimmune disease expression, region-specific data are essential.

This study aims to evaluate serum vitamin D levels in patients with SLE at Mandalay General Hospital and investigate their association with disease activity, as measured by the SLAM score. We hypothesized that vitamin D deficiency is more prevalent among SLE patients with active disease compared to those in remission. Establishing this relationship may support the role of vitamin D as a modifiable biomarker in SLE management and provide direction for potential supplementation strategies.

## Materials and methods

This hospital-based, cross-sectional analytical study was conducted at Medical Units I, II, and III of Mandalay General Hospital, Myanmar, over one year from January 1, 2019, to December 31, 2019. Mandalay General Hospital is one of the largest tertiary care and teaching hospitals in the country, serving as a referral center for complex medical conditions, including systemic autoimmune diseases such as SLE, which is known to have high prevalence and significant disease burden in vitamin D-deficient populations.

The study was conducted in accordance with ethical principles outlined by the institutional review board. Ethical approval was obtained from the Research and Ethics Committee of the relevant institution. Informed written consent was obtained from each participant after the study objectives, procedures, potential benefits, and risks were clearly explained. Participation was entirely voluntary, and confidentiality was maintained through coded data. Patients retained the right to withdraw from the study at any point without consequence. No financial incentives or additional procedures were involved.

The sample size was calculated using Stata version 13, based on data from a previous study by Amital et al., which demonstrated an inverse relationship between serum 25-hydroxyvitamin D levels and disease activity in patients with SLE [1]. Assuming a power of 80%, a confidence level of 95%, and a mean serum vitamin D level of 36 ± 5.7 ng/mL in the general population compared to 20.1 ± 17.0 ng/mL in SLE patients, the minimum required sample size was 45. To enhance statistical power and ensure data validity, 60 patients were recruited.

All eligible patients diagnosed with SLE and attending the three internal medicine units of Mandalay General Hospital during the study period were consecutively enrolled. Inclusion criteria included fulfillment of four or more of the 1982 revised classification criteria for SLE established by the American College of Rheumatology. Exclusion criteria included pregnancy, recent use (within six months) of vitamin D or calcium supplementation, regular use of sunscreen products, chronic liver or kidney disease, ongoing anti-tuberculosis therapy for more than two weeks, or use of enzyme-inducing medications such as phenobarbitone or carbamazepine.

Clinical data were collected using a standardized pro forma capturing demographic information, detailed clinical history, and physical examination findings. The complete data collection form used in this study is provided in Table 9. Disease activity was assessed using the Systemic Lupus Activity Measure (SLAM) score on the day of blood sampling. Patients were classified into active and inactive disease groups according to their SLAM scores [12]. To maintain consistency in clinical assessments, standardized training protocols were used for all investigators involved.

Laboratory investigations included collection of 5 mL of venous blood under aseptic conditions, avoiding the use of a tourniquet. Samples were centrifuged at 2,000 rpm for 20 minutes, stored at −20°C, and transported in a cold chain to the National Health Laboratory, Mandalay. Serum 25-hydroxyvitamin D [25(OH)D] levels were measured using an enzyme-linked immunosorbent assay (ELISA). Routine investigations, including complete blood count, erythrocyte sedimentation rate (ESR), serum creatinine, urine microscopy, chest X-ray, and electrocardiogram, were performed using calibrated and quality-controlled equipment at the hospital's central laboratory.

Data were manually coded and entered into Microsoft Excel 2007 (Microsoft Corporation, Redmond, USA) and analyzed using Stata version 11 on Windows 7. Quantitative variables were summarized as mean ± standard deviation (SD), while categorical variables were reported as frequencies and percentages. Between-group comparisons of continuous variables were conducted using the independent Student’s t-test, and Fisher’s exact test was used for categorical data. A p-value < 0.05 was considered statistically significant. Odds ratios (ORs) with 95% confidence intervals (CIs) were computed for relevant categorical associations [12]. The relationship between serum vitamin D levels and SLE disease activity was evaluated using two complementary methods: categorical comparison with Fisher’s exact test-revealing a significant association (p = 0.004) and Pearson’s correlation coefficient, which showed a moderate inverse correlation (r = −0.51, p < 0.001) between continuous SLAM scores and vitamin D levels [13].

## Results

A total of 60 patients diagnosed with SLE were enrolled in this study. The mean age was 25.77 ± 7.98 years (range: 13-45 years), with the highest proportion of patients (43.33%, n = 26) in the 20-29-year age group, followed by 26.67% (n = 16) below 20 years, 25.00% (n = 15) between 30-39 years, and 5.00% (n = 3) above 40 years (Table 1). The study population demonstrated a strong female predominance (98.33%, n = 59), with only one male patient (1.67%) (Table 2). Due to this marked gender imbalance, statistical analyses were primarily focused on female participants; the single male case was not analyzed separately but acknowledged as a limitation related to gender bias.

**Table 1:** Age Distribution of SLE Patients SLE: Systemic lupus erythematosus

Age (years)	No. of Patients	Percentage (%)
<20	16	26.67
20–29	26	43.33
30–39	15	25
>40	3	5
Total	60	100

**Table 2:** Sex distribution of SLE patients SLE: Systemic lupus erythematosus

Sex	No. of Patients	Percentage (%)
Male	1	1.67
Female	59	98.33
Total	60	100

Among the 60 SLE patients, 85% (n = 51) exhibited vitamin D deficiency (serum 25(OH)D ≤ 30 ng/mL), while only 15% (n = 9) had sufficient levels (<30 ng/mL). The mean serum vitamin D concentration was 16.82 ± 11.24 ng/mL, reflecting a high prevalence of insufficiency in this study (Table 3).

**Table 3:** Vitamin D Status in SLE Patients SLE: Systemic lupus erythematosus

Serum Vitamin D Level	No. of Patients	Percentage (%)
≤30 ng/mL (Deficient)	51	85
>30 ng/mL (Sufficient)	9	15
Total	60	100
Mean ± SD	16.82 ± 11.24	

Among clinical features, anemia was the most frequently observed manifestation (56.67%, n = 34), followed by skin rash (33.33%, n = 20) and mucosal ulcers (16.67%, n = 10) (Table 4).

**Table 4:** Clinical Manifestations of SLE Patients SLE: Systemic lupus erythematosus

Clinical Manifestations	Frequency	Percentage (%)
Anemia	34	56.67
Skin Rash	20	33.33
Mucosal Ulcer	10	16.67
Arthritis	10	16.67
Nephritis	7	11.67
Vasculitis	7	11.67
Serositis	7	7
Neuropsychiatric Lupus	3	5

Hematological abnormalities were common among the 60 SLE patients. Mean hemoglobin was 9.49 ± 1.59 g/dL, indicating anemia. White blood cell count was mildly reduced (5.32 ± 3.02 ×10³/µL), with a mean lymphocyte count of 1.38 ± 0.67 ×10³/µL, consistent with lymphopenia. The ESR was markedly elevated (82.53 ± 23.85 mm/hr), reflecting systemic inflammation. Mean serum creatinine was 96.4 ± 28.48 µmol/L, suggesting preserved renal function in the majority of patients (Table 5).

**Table 5:** Laboratory Parameters in SLE Patients SLE: Systemic lupus erythematosus

Laboratory Parameters	Mean ± SD
Hemoglobin (g/dL)	9.49 ± 1.59
WBC (×10³/µL)	5.32 ± 3.02
Lymphocyte (×10³/µL)	1.38 ± 0.67
Platelet (×10³/µL)	184.3 ± 71.08
ESR (mm/hr)	82.53 ± 23.85
Serum Creatinine (µmol/L)	96.4 ± 28.48

Disease activity was assessed using the SLAM score. Active disease was observed in 66.67% (n = 40) of patients, while 33.33% (n = 20) were classified as inactive (Table 6).

**Table 6:** Disease Activity Stratification by SLAM Score SLAM: Systemic Lupus Activity Measure

SLAM Status	No. of Patients	Percentage (%)
Active SLE	40	66.67
Inactive SLE	20	33.33
Total	60	100

Furthermore, vitamin D deficiency was more prevalent among those with active disease (95%, n = 38) than in those with inactive disease (65%, n = 13), a difference that was statistically significant (p = 0.004) (Table 7).

**Table 7:** Association Between Vitamin D Deficiency (≤30 ng/mL) and SLE Disease Activity (Fisher’s Exact Test) SLE: Systemic lupus erythematosus

Vitamin D level	Active SLE		Inactive SLE		Total	Fisher Exact (p value)
	n	%	n	%		
≤ 30 ng/ml	38	95.00	13	65.00	51(160)	0.004
>30 ng/ml	2	5.00	7	35.00	9(40)	
Total	40	100.00	20	100.00	60(200)	

These findings demonstrate a high prevalence of vitamin D deficiency in SLE patients, particularly among those with active disease, suggesting a potential role of vitamin D in disease modulation. The correlation between serum vitamin D levels and SLAM disease activity scores is shown in Table 8. The strong inverse relationship between vitamin D levels and disease activity warrants further investigation into therapeutic vitamin D supplementation in SLE management.

**Table 8:** Correlation Between Serum Vitamin D Levels and SLAM Score Using Pearson’s r SLAM: Systemic Lupus Activity Measure; ng/mL: Nanograms per Milliliter. Pearson’s r is the correlation coefficient indicating the strength and direction of a linear relationship between two variables.

Variable 1	Variable 2	Pearson’s r	p-value	Interpretation
Vitamin D (ng/mL)	SLAM Score	−0.51	< 0.001	Moderate inverse correlation

## Discussion

SLE is a complex autoimmune disorder characterized by multifaceted clinical presentations and a multifactorial etiology involving genetic, environmental, and immunological factors. Our study demonstrates a significant inverse association between vitamin D deficiency and disease activity in SLE patients within a Myanmar cohort, a population where such data have been scarce. Patients with active disease exhibited notably lower serum vitamin D levels compared to those with inactive disease, consistent with previous findings by Amital et al. and Borba et al. [1,3], supporting the hypothesis of vitamin D as an immunomodulator in autoimmune conditions.

The demographic profile of our cohort-marked female predominance (98.33%) and peak incidence in reproductive-age women (20-29 years) aligns with global SLE epidemiology. Anemia was the predominant clinical manifestation (56.7%), followed by cutaneous and musculoskeletal symptoms, paralleling other Asian cohorts and suggesting both shared and regional disease characteristics [10]. The high prevalence of vitamin D deficiency (mean 16.82 ± 11.24 ng/mL) likely results from multiple interacting factors: photosensitivity leading to deliberate sun avoidance, cultural clothing practices limiting skin exposure to sunlight, and possible genetic variants affecting vitamin D metabolism. Additionally, nutritional deficiencies may play a role, given the limited dietary vitamin D sources in the region [11,12].

Biologically, vitamin D is known to influence immune regulation through modulation of dendritic cell maturation, T-cell differentiation, and cytokine production, promoting a shift from pro-inflammatory Th1 and Th17 responses towards regulatory T-cell phenotypes. This immunomodulatory capacity can theoretically attenuate autoimmune responses, reducing disease activity in conditions such as SLE. However, the cross-sectional nature of our study restricts causal inferences. An important alternative explanation is reverse causality: active SLE manifestations, particularly photosensitive rashes, may lead patients to limit sun exposure, thereby lowering endogenous vitamin D synthesis. Thus, low vitamin D levels could be a consequence rather than a cause of increased disease activity [14].

Medication confounding is another critical consideration. Glucocorticoids, commonly prescribed in SLE, can impair vitamin D metabolism by increasing catabolism and reducing intestinal absorption, potentially exacerbating deficiency. Our study did not control for cumulative steroid dosage or duration, which could influence serum vitamin D levels and confound the observed association. Future studies should rigorously adjust for medication effects to clarify these interactions.

From a practical standpoint, clinicians managing SLE patients in Myanmar should consider routine screening for vitamin D deficiency, especially in those presenting with active disease or known risk factors such as photosensitivity and glucocorticoid use. Given the high prevalence observed, annual screening may be prudent, with more frequent monitoring during periods of increased disease activity or therapy adjustment. Supplementation thresholds should ideally aim to maintain serum 25(OH)D levels above 30 ng/mL, consistent with international guidelines for bone and immune health, though region-specific recommendations may evolve with further research.

While our findings reinforce the potential importance of vitamin D in SLE management, they should be interpreted with caution. The study’s design is associative, not causative, and limited by sample size and lack of control for dietary intake, medication regimens, and genetic polymorphisms affecting vitamin D metabolism [15]. Longitudinal and interventional studies are necessary to determine whether correcting the deficiency can mitigate disease activity or improve clinical outcomes.

## Conclusions

This study reveals a significant inverse association between serum vitamin D levels and disease activity in SLE patients, with a high prevalence of deficiency, particularly among those with active disease. These results suggest a plausible immunomodulatory role of vitamin D in the pathogenesis and exacerbations of SLE but do not establish causality. Considering the modifiable nature of vitamin D status, routine monitoring and appropriate supplementation may be beneficial components of comprehensive SLE care in Myanmar.

To further elucidate vitamin D’s therapeutic potential, randomized controlled trials of vitamin D supplementation in deficient SLE patients are warranted. Such trials should evaluate optimal dosing, effects on disease activity, flare prevention, and quality of life. Meanwhile, clinicians should adopt a balanced approach, integrating vitamin D assessment into routine care while recognizing that supplementation is one of multiple factors influencing complex autoimmune disease trajectories.

## Appendices

SLE patient data collection form

**Table 9:** SLE Patient Data Collection Form SLAM: Systemic Lupus Activity Measure; 25(OH)D: 25-Hydroxyvitamin D; Hb: Hemoglobin; WBC: White Blood Cell; ESR: Erythrocyte Sedimentation Rate; Anti-dsDNA: Anti-Double-Stranded DNA. All units and categories are specified to facilitate reproducibility and clarity.

Patient Information		
ID	Unique patient identifier	Alphanumeric
Age	Patient's age in years	Numerical (years)
Sex	Patient's biological sex	Female, Male
Date	Date of data collection	DD/MM/YYYY
Disease Activity		
SLAM Score	Systemic Lupus Activity Measure score	Numerical
Status	Disease activity status based on SLAM score	Active (SLAM > 6), Inactive (SLAM ≤ 6)
Vitamin D Status		
25(OH)D Level	25-hydroxyvitamin D level	Numerical (ng/mL)
Status	Vitamin D status based on 25(OH)D level	Deficient (≤ 30), Sufficient (> 30)
Clinical Features	(Check all present)	Yes/No
Anemia	Presence of anemia	Yes/No
Malar rash	Presence of malar rash	Yes/No
Discoid rash	Presence of discoid rash	Yes/No
Oral/Nasal ulcers	Presence of oral/nasal ulcers	Yes/No
Arthritis	Presence of arthritis	Yes/No
Renal involvement	Presence of renal involvement	Yes/No
Serositis	Presence of serositis	Yes/No
Neuropsychiatric symptoms	Presence of neuropsychiatric symptoms	Yes/No
Laboratory Results		
Hb	Hemoglobin level	Numerical (g/dL)
WBC	White blood cell count	Numerical (×103/μL)
Lymphocytes	Lymphocyte count	Numerical (×103/μL)
Platelets	Platelet count	Numerical (×103/μL)
ESR	Erythrocyte sedimentation rate	Numerical (mm/hr)
Creatinine	Creatinine level	Numerical (μmol/L)
Anti-dsDNA	Anti-double-stranded DNA antibody status	Positive, Negative
Current Medications		
Prednisone	Prednisone dosage	Numerical (mg/day), or No
Hydroxychloroquine	Use of Hydroxychloroquine	Yes/No
Immunosuppressants	Specific immunosuppressant medication(s)	Text entry for specific drug, or No
None	No current medications	Yes/No
